# Management of Esophageal Carcinoma Associated with Cirrhosis: A Retrospective Case-Control Analysis

**DOI:** 10.1155/2009/173421

**Published:** 2009-12-22

**Authors:** Florence Trivin, Eveline Boucher, Elodie Vauléon, Isabelle Cumin, Elisabeth Le Prisé, Odile Audrain, Jean-Luc Raoul

**Affiliations:** ^1^Department of Medical Oncology, Centre Eugène Marquis, CS 44229, 35042 Rennes Cedex, France; ^2^Department of Oncology, Centre Hospitalier de Bretagne Sud, 56100 Lorient, France; ^3^Department of Radiotherapy, Centre Eugène Marquis, CS 44229, 35042 Rennes Cedex, France

## Abstract

*Objectives*. Esophageal carcinoma and cirrhosis have the overlapping etiologic factors. *Methods*. In a retrospective analysis conducted in 2 Breton institutions we wanted to asses the frequency of this association and the outcome of these patients in a case-control study where each case (cirrhosis and esophageal cancer) was paired with two controls (esophageal cancer). *Results*. In a 10-year period, we have treated 958 esophageal cancer patients; 26 (2.7%) had a cirrhosis. The same treatments were proposed to the 2 groups; cases received nonsignificantly different radiation and chemotherapy dose than controls. Severe toxicities and deaths were more frequent among the cases. At the end of the treatment 58% of the cases and 67% of the controls were in complete remission; median and 2-year survival were not different between the 2 groups. All 4 Child-Pugh B class patients experienced severe side effects and 2 died during the treatment. *Conclusions*. This association is surprisingly infrequent in our population! Child-Pugh B patients had a dismal prognosis and a bad tolerance to radiochemotherapy; Child-Pugh A patients have the same tolerance and the same prognosis as controls and the evidence of a well-compensated cirrhosis has not modified our medical options.

## 1. Introduction

The incidence of oesophageal cancer is high in France and particularly in Brittany [1]. Alcohol and smoking are the main etiological factors of squamous-cell carcinoma [2], the most frequent type of oesophageal cancer in our region. This alcohol-smoking combination also predisposes to cirrhosis, with alcoholic cirrhosis being frequent and smoking increasing the severity of liver diseases [3]. The possible association of oesophageal cancer and cirrhosis worsens the prognosis and raises serious therapeutic problems. Surgery is often contraindicated or associated with a high morbidity [4, 5], but to our knowledge, no data are currently available concerning whether these patients could benefit from a specific medical treatment. It is also unknown whether their prognosis is different from patients with cancer of the oesophagus but without cirrhosis. We therefore analyzed retrospectively the frequency of this association in our patient population in order to examine treatment efficacy and develop a therapeutic proposal.

## 2. Materials and Methods

This retrospective study included all patients treated for cancer of the oesophagus between January 1, 1993 and December 31, 2002 at two cancer centers in Brittany, the *Centre Eugène-Marquis* (CEM) (the regional comprehensive cancer center located in the city of Rennes) and the *Centre Hospitalier de Bretagne Sud *(CHBS) (located in the city of Lorient). We retained for analysis all patients with the diagnosis of both cancer of the oesophagus and cirrhosis. Medical files were re-examined to confirm the diagnosis of associated cirrhosis. Each patient in this group (case group) was matched with two patients with cancer of the oesophagus but without cirrhosis (control group) treated at the CEM during the same period. Patients were matched for gender, age (±5 years), TNM stage, tumor localization (upper, middle, lower oesophagus), histological type, and period of treatment (±2 years).

The following data were noted for each patient: gender, age, alcohol-tobacco consumption, histology, tumor localization, TNM stage (assessed with computed tomography or endoscopic ultrasonography), type of treatment (surgery, radiotherapy-chemotherapy, chemotherapy, other), radiation dose, total chemotherapy dose administered (expressed in percent of theoretical dose per body surface area), treatment adaptations, toxicity (CTC-NCI classification, version 2.0), tumor stage at treatment end, recurrence and date of death or last follow-up; information, etiology, circumstance of discovery, Child-Pugh score, in patients with cirrhosis.

Quantitative variables (survival, radiotherapy dose) were expressed as median or mean ± standard deviation (SD). Qualitative variables were expressed as number (*n*) and percentage (%). Overall survival was determined from the date of diagnosis to the date of death or last follow-up information. For patients who achieved complete remission, recurrence-free survival was determined from diagnosis to date of progression or last follow-up information. Complete remission was defined as absence of suspected oesophageal lesions at endoscopy, normal histology of systematic biopsy specimens, and absence of tumor aspect on the computed tomography (CT) performed approximately three months after the end of the radiation protocol. Survival curves were established using the Kaplan-Meier method and compared with the log-rank test. *P* < .05 was considered significant. The chi-square test with Yates correction as appropriate was applied to compare qualitative variables.

## 3. Results

From January 1, 1993 to December 31, 2002, 671 and 287 patients were treated for oesophageal cancer at the CEM and CHRBS, respectively. For 26 of these 958 patients (3%), the summary diagnosis mentioned “cirrhosis.” The tumor staging in these 26 patients (21 men, 5 women, mean age 58.9 ± 7.7 years) was IIA (*n* = 4), IIB (*n* = 3), III (*n* = 18), IV (*n* = 1). All 26 had alcoholic cirrhosis which was known before the diagnosis of oesophageal cancer in 11 (38.5%). Histological proof of cirrhosis was available for seven patients. The diagnosis was based on the presence of oesophageal varices (often associated with a history of hepatic decompensation with edema and ascites) in sixteen patients and on a previous history of decompensation with edema and ascites in three. The Child-Pugh classification was A in 22 and B in 4. Among these 4 Child B patients none had ascites.

### 3.1. Treatments Administered and Toxicity in the Case Group

Among the 26 patients in the case group, none had been operated, 25 were given combined radiochemotherapy using the Herskovic protocol [6] in 16 and another protocol in 9; in these 9 cases the chemotherapy protocol also used 5FU and CDDP following an LV5FU2 regimen associated with CDDP at the dose of 50 mg/m² every two weeks. One patient with metastatic disease was given chemotherapy (5FU and CDDP) alone. Compared with the theoretical dose for the prescribed Herskovic protocol, the dose delivered was 75 ± 25% for CDDP and 59 ± 32% for 5FU (partly because the 5FU dose was decreased to 600 mg/m² from the first session for 14 patients). Median radiation dose delivered was 54 Gy (range 12–65 Gy). Haematologic and digestive toxic effects are summarized in Table 1. Other severe complications were noted in 9 patients (35%): hepatic decompensation in 5 (leading to death in 2), acute lower limb ischemia following chemotherapy infusion in 1 patient, hepatic encephalopathy in 1 patient (death), hematemesis in 1 patient (death), hemoperitoneum subsequent to gastric invasion in 1 patient (death). In all, 5 patients died during treatment; four deaths (15.5%) were related to cirrhosis. Chemotherapy delivery had to be modified because of toxic effects in 9 patients (34.5%).

At the end of treatment, 5 patients (19%) including two classified Child B had died, 15 patients (58%) were in complete remission, and 6 (23%) had active disease. Median survival was 10 months (range 1–61 months). Overall 2-year survival was 28 ± 9%. Median recurrence-free survival was 13 months; the 2-year recurrence-free survival rate was 42 ± 14%.

### 3.2. Comparison with the Control Group

The matched control group showed no significant difference from the case group but there were fewer (NS) alcoholic patients in the control group. There was no significant difference between the groups for choice of treatment: 44 of the 52 control patients were given combined radiochemotherapy (with a Herskovic protocol for 28 and an LV5FU2-CDDP ergimen for the others) and 4 were given chemotherapy alone, but 4 patients in the control group underwent surgery. The radiation dose delivered was 50 Gy (range 30–65 Gy) and the percentage of the theoretical dose was 73 ± 25% for CDDP and 67 ± 25% for 5FU. This was not significantly different from the case group. Regarding the classical toxic effects of chemotherapy (Table 1), there were more cases of grade 3-4 mucitis in the control group (16/48 versus 2/26 in the case group, *P* = .05). There was not however any significant difference for hematological, gastrointestinal, or renal complications. Nine patients (16%) in the control group presented other severe complications: major degradation of general status in 1, severe pneumonia in 3, septic shock in 2, abdominal wall infection on a jejunostomy orifice in 1, rhythm disorder in 1, and esophagotracheal fistulization leading to death in 3 patients (6%). Surprisingly, the proportion of patients who developed severe grade 3-4 or fatal complications was not significantly different between the two groups (Table 1): 11/26 in the case group and 21/48 in the control group. There were fewer deaths in the control group (*n* = 3, 6% versus *n* = 5, 19% in the case group) but the difference was not significant.

In the control group, at the end of treatment 3 patients had died, 35 (67%) were in complete remission, and 14 had active disease. There was no significant difference (*P* = .18) in terms of outcome after treatment between the two groups.

**Figure 1:**
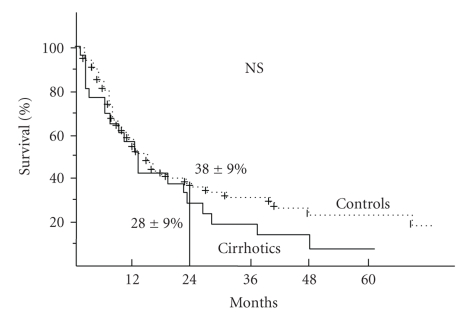
Overall survival in the control group (esophageal cancers) and in the case group (esophageal cancer in cirrhotic patients): no significant difference between the two groups.

Overall survival was not significantly different between the two groups (Figure 1). Median survival was 10 months in the case group and 14 months in the control group. The overall 2-year survival rate was 38 ± 9% in the control group versus 28 ± 9% in the case group (NS).

There was no significant difference in recurrence-free survival between the two groups. Median recurrence-free survival was 14 months in the control group and the 2-year recurrence-free survival rate was 37 ± 7% in the control group.

### 3.3. Comparison by Child-Pugh Score

**Figure 2:**
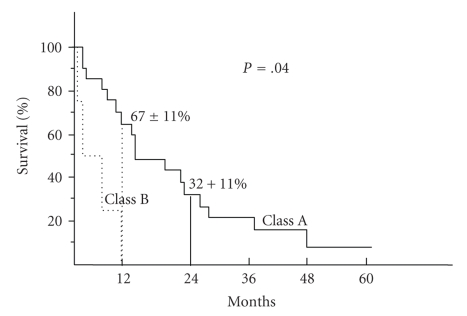
Overall survival in the cirrhotic patients with esophageal carcinoma: significant difference between class A and class B patients.

Despite the small number of Child B patients, there was a marked difference in survival between Child A and Child B patients (*P* = .04) (Figure 2). The 1-year survival was 67% for Child A patients and 0% for Child B patients. This difference in prognosis was not related to age or major difference in tumor severity. There was however a difference in terms of treatment tolerance. Tolerance was poorer in Child B patients, all treated with combined radiochemotherapy. All developed a major complication: death due to liver failure with edema and ascites, death with hemiperitoneum, grade 4 thrombopenia, grade 4 neutropenia. The 4 Child B patients died within one year of diagnosis, two during treatment, one at six months from disease progression, and one at 10 months from an unknown cause despite complete remission.

## 4. Discussion

This small series of patients highlights several interesting points. The frequency of cirrhosis was surprisingly low among our patients with cancer of the oesophagus. The treatments proposed were very similar for patients with and without cirrhosis, although surgery was never proposed for cirrhotic patients. Toxic effects were particularly severe in cirrhotic patients but were generally related to their liver disease; there was no difference in the rate of “classical” complications, particularly hematological disorders or mucitis, which was less frequent in cirrhosis patients despite similar treatments (radiation dose and chemotherapy). Treatment efficacy and survival were not different between the groups. Toxicity was however a major problem in Child B patients whose prognosis was much less favorable.

In our case population, the proportion of cirrhotic patients (3%) was much lower than generally reported. Two studies from Japan [5] and Italy [7] have reported the cirrhosis-oesophageal cancer association in 7% and 14% of patients. In a French autopsy report [8], liver disease (alcoholic cirrhosis, acute alcoholic hepatitis, hepatic fibrosis) was found in 18% of patients who had died from oesophageal cancer. There could be several explanations for this apparent discrepancy. First, gastroenterologists, who referred all of our patients, probably preferentially selected cirrhotic patients for endoscopic treatment or therapeutic abstention. This could certainly explain the absence of Child C patients. Esophagogastroduodenal endoscopy was performed in all patients, enabling detection of oesophageal varices if the scope could be passed through the stricture. Prothrombin time and other laboratory tests were also available for all patients, but no attempt was made to systematically search for purely histological forms of cirrhosis. This probably led to a clinical underestimation of cirrhosis in comparison with surgical series where there is at least a macroscopic assessment of liver disease. Age at disease onset, as well as favoring factors, is similar for the two conditions under consideration [9]. Certain protective factors (different metabolic mechanism of carcinogenesis in the presence of cirrhosis?), or on the contrary, phenotypic or genotypic configurations possibly aggravating toxic effects could also be involved. A recent report from China demonstrated differences in the frequency of ADH2 and ALDH2 genes between alcoholic patients who developed alcoholic cirrhosis and those who developed oesophageal cancer [10] but no similar data are currently available for French patients. One other Breton team [11] has examined the genetic polymorphism of two P450 cytochromes (CYP2EI and CYP1A1) among alcoholic patients with diverse complications (including cirrhosis and oesophageal cancer) and control subjects, but was unable to demonstrate a significant difference. The hypothesis of differential toxicity of alcohol and tobacco warrants further exploration. But such discrepancies are also found in epidemiological studies. An Italian study [12] demonstrated an increased risk of oesophageal cancers among cirrhotic patients (odds ratio of 2.6), but a Danish cohort study [13] failed to demonstrate such an association. The proportion of female patients in our case group (20%) was higher than in the overall population of patients treated for oesophageal cancer in our institution (9%). Here again the question concerns the underlying source of the difference: genetic effect, type of alcoholism?

Beyond the fact that surgery was not performed for any of the cirrhotic patients, it is clear that the proposed therapeutic options were quite similar for all patients. Radiochemotherapy, generally with a Herskovic protocol [6], was proposed for the large majority of patients in both groups. Toxicity was not significantly worse in the case group, patients in this group even presenting fewer episodes of mucitis! This difference cannot be explained by an excess of mucosal toxicity in our control population since the proportion was similar to that reported by Herskovic et al. [6]. It might be explained by the lower 5FU dose administered in our case group patients starting from the first cycle, in line with the conclusions of Bleiberg et al. [14] who demonstrated less toxicity (but comparable efficacy) in patients given palliative treatment using CDDP alone compared with 5FU-CDDP. Despite the initial hypersplenism subsequent to the portal hypertension, our case group patients did not present a significantly increased rate of hematological complications. This is probably because these patients had a peripheral rather than central hematological disorder. We did not note any renal toxicity, but fluid infusion did lead to decompensation with edema and ascites in 5 patients. This suggests that CDDP doses should be fractionated for this type of patient in order to limit fluid overload or that carboplatin should be used in such high-risk patients.

Despite the absence of a significant difference between the groups, there were more deaths in the case group (19%) than in the control group (6%). Most of the deaths were related to complications of cirrhosis and occurred in patients with Child B disease. This very poor tolerance to treatment is probably sufficient to prefer radiation alone for Child B patients instead of the classical radiochemotherapy protocol.

The proportion of patients who achieved compete remission at the end of treatment was similar in the two groups, a result which is coherent with the identical radiation and chemotherapy doses delivered. The overall and recurrence-free survivals were also equivalent for the two groups, a result which is coherent with the natural history of the two diseases: initial overmortality in cirrhotic patients masked by the dismal prognosis of oesophageal cancer; absence of cirrhosis-related overmortality after chemoradiotherapy since the only surviving cirrhosis patients were Child A.

## 5. Conclusion

We found that the proportion of patients with oesophageal cancer who have cirrhosis is low despite similar favoring factors in our population (population without any alcohol flushing response) [15]. Patients with oesophageal cancer and well compensated cirrhosis (Child A) tolerate radiochemotherapy as well as patients with oesophageal cancer alone and respond similarly in terms of antitumor effect and survival. Patients with more severe liver disease (Child B) develop serious cirrhosis-related complications contraindicating a classical radiochemotherapy protocol. Thus for patients with cancer of the oesophagus and cirrhosis, it would appear advisable to propose a classical regimen for Child A patients and a less aggressive treatment (radiotherapy, endoscopic treatment) for Child B patients.

## Figures and Tables

**Table 1:** Chemotherapy toxicity (grade 3/grade 4) in oesophageal cancer patients with (case group) or without liver cirrhosis (control group); severe toxicity corresponds to patient with at least one grade 3 or 4 toxicity.

	Case group *n* = 26	Control group *n* = 48	*P*
Platelets grade 3/4	2/1	4/1	NS
PMN grade 3/4	5/2	4/4	NS
Mucitis grade 3/4	2/0	12/4	.05
Vomiting grade 3/4	2/0	0/0	NS
Other: severe	9	9	NS
Death during treatment	5	3	NS
Severe toxicity: no/yes	11/15	21/27	NS
